# Supplementary material for: Effects of an Amphiphilic Micelle of Diblock Copolymer on Water Adsorption of Cement Paste
Source: Materials (Basel). 2023 Mar 9;16(6):2190. doi: 10.3390/ma16062190 (PMC10056578; doi:10.3390/ma16062190)
Supplement: Supplementary file 1 [file materials-16-02190-s001.zip › materials-2253178-supplementary.pdf]

Effects of an Amphiphilic Micelle of Diblock Copolymer on Water Adsorption of Cement Paste

Lei Dong [1,*], Fei Meng [1], Pan Feng [1,*], Qianping Ran [1,2], Chonggen Pan [3] and Jianming He [4]

1 School of Materials Science and Engineering, Southeast University, Nanjing 211189, China
2 State Key Laboratory of High-Performance Civil Engineering Materials, Jiangsu Sobute New Materials Co., Ltd., Nanjing 211103, China
3 School of Civil Engineering and Architecture, Ningbo Tech University, Ningbo 315100, China
4 Ningbo Construction Guangtian Component Co., Ltd., Ningbo 315100, China
* Correspondence: leidong@seu.edu.cn (L.D.); pan.feng@seu.edu.cn (P.F.)

Table S1. Chemical composition (wt%) of ordinary Portland cement (PO 42.5) used in this study.

Composition	SiO_2	Al_2O_3	Fe_3O_4	CaO	MgO	K_2O	Na_2O	SO_3	Others	LOI
Content	21.60	4.35	2.95	63.81	1.76	0.51	0.16	2.06	1.61	1.19

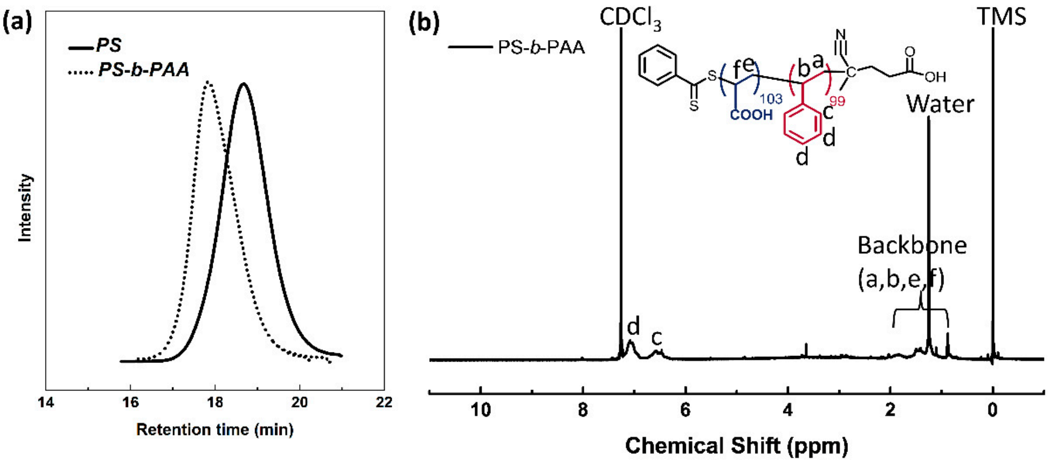

Figure S1. (a) SEC chromatography of PS-homopolymer with active RAFT groups and diblock copolymer PS-*b*-PAA following the sequential polymerization of acrylic acid monomers and (b) ^{1}H NMR spectrum of synthesized PS-*b*-PAA in solvent chloroform -*d*.

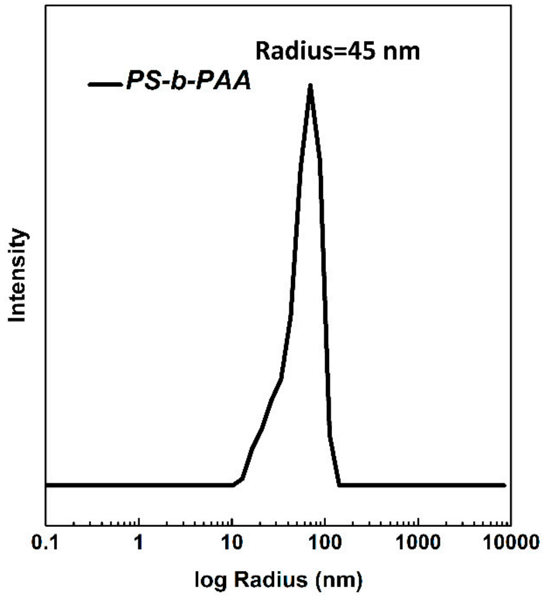

Figure S2. The measured particle size distribution from dynamic light scattering.

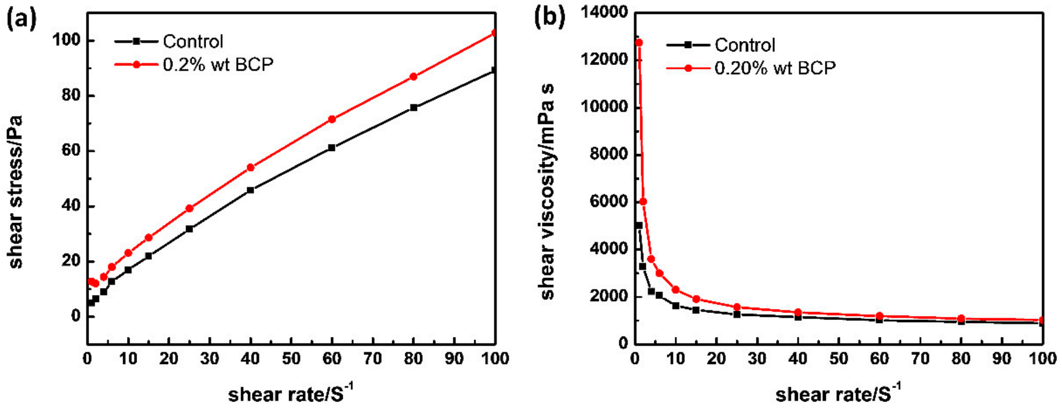

Figure S3. (a) The measured shear stress vs. shear rate and (b) shear viscosity vs. shear rate curves for studied cement pastes at the initial time.

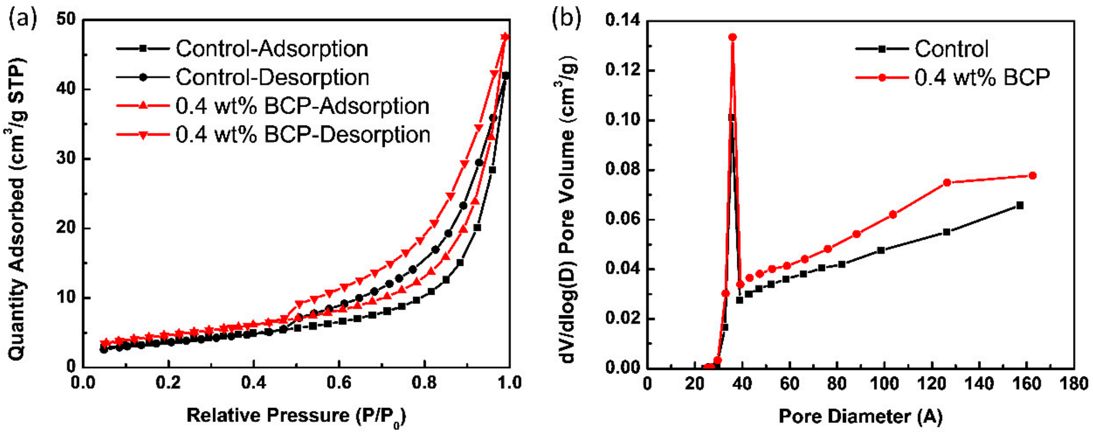

Figure S4. (a) BET surface area and (b) pore size distribution curves of control and BCP doped cement pastes samples at 28-days.

Table S2. Basic parameters of pore structure estimated from BET characterization.

Sample		Cumulative Surface Area of Pores (m²/g)	Cumulative Volume of Pores (cm³/g)	Average Pore Diameter (Å)
Adsorption	Control	14.592	0.023257	63.751
	0.4wt% BCP	18.432	0.030653	66.524
Desorption	Control	20.4308	0.036202	70.876
	0.4wt% BCP	25.5076	0.045692	71.653
